# Use of Fermented Black Tea (*Camellia sinensis*) Factory Wastes in Standard Rat Diets

**DOI:** 10.3390/vetsci12050451

**Published:** 2025-05-08

**Authors:** Buğra Genç, Nilüfer Kuruca, Gül Fatma Yarım, Tolga Güvenç, Emre Özan, Bahadır Müftüoğlu, Tayfun İde, Aşkın Nur Derinöz Erdoğan, Serdar Odacı

**Affiliations:** 1 Department of Laboratory Animals, Faculty of Veterinary Medicine, Ondokuz Mayis University, 55100 Samsun, Türkiye; emre.ozan@omu.edu.tr (E.Ö.); bahadir.muftuoglu@omu.edu.tr (B.M.); 2 Department of Pathology, Faculty of Veterinary Medicine, Ondokuz Mayis University, 55100 Samsun, Türkiye; nilufer.kuruca@omu.edu.tr (N.K.); tguvenc@omu.edu.tr (T.G.); 3 Department of Biochemistry, Faculty of Veterinary Medicine, Ondokuz Mayis University Samsun, 55100 Samsun, Türkiye; gulyarim@omu.edu.tr; 4 ARDEN Research an Experiment, Ankara 06170, Türkiye; tayfunide2003@yahoo.com (T.İ.); info@ardenarastirma.com (A.N.D.E.); 5 Samsun Veterinary Control Institute, Republic of Türkiye Ministry of Agriculture and Forestry, Samsun 55200, Türkiye; serdar.odaci@tarimorman.gov.tr

**Keywords:** antioxidant, diet, fermentation, rat, tea factory waste

## Abstract

Although there are studies on the use of different tea extracts in animal diets, there are no studies on the use of fermented black tea factory production waste (FTFW) in rodent diets. This study aims to evaluate the effects of FTFW, considered an environmentally hazardous waste, in standard rat diets regarding nutritional, histopathological, and biochemical parameters. In this study, 40 male Wistar albino rats (70 days old, with a live weight of 200–250 g) were divided into four groups, each containing 10 rats. They were fed standard rat diets for 63 days, with varying amounts (0%, 3%, 5%, and 10%) of tea production waste. At the end of the experiment, biochemical analysis of blood and histopathological analysis of all organs were performed. There was no significant difference between the groups in terms of body weight, internal organ weights, or serum biochemical parameters (*p* > 0.05). No pathological findings were observed in any of the groups. The group receiving 10% tea waste, which had the highest total polyphenol level (1.42 ppm), also showed the highest serum total antioxidant status (TAS) (*p* < 0.05). In contrast, this group had the lowest total oxidant status (TOS) and oxidative stress index (OSI) levels (*p* < 0.05). The study concluded that FTFW can be included in the pellet diet of rats without changing their general health status and that a potential environmental pollutant can be used in rat diets.

## Simple Summary

This study investigates fermented black tea factory production waste (FTFW) in rat diets, which has not been explored before. The goal is to assess the effects of FTFW, a waste considered environmentally harmful, on nutrition, health, and biochemical parameters in rats. Forty male rats were divided into four groups and fed diets with different amounts of tea waste (0%, 3%, 5%, and 10%) for 63 days. After the experiment, blood and organ samples were analyzed. The results showed that the caffeine in the tea waste was mostly removed during pellet production, and there were no significant differences in body weight, organ weights, or blood parameters between the groups. Additionally, no health issues or behavioral changes were observed. The group fed the highest amount of tea waste (10%) showed the best antioxidant levels, with lower oxidant levels and oxidative stress. Unlike other studies on tea extracts, no negative effects were found with FTFW. The study suggests that FTFW can be safely added to rat diets without harming their health, offering a low-cost, environmentally friendly way to turn waste into a beneficial product.

## 1. Introduction

The countries with the highest tea production and consumption worldwide are China, India, Kenya, and Türkiye, respectively. The global tea production volume was 3,190,000 tons in 2010, exceeding 6 million tons in 2023. It is projected to surpass 7.5 million tons by 2025 [1]. Teas are classified as green, black, and oolong based on the oxidation and fermentation techniques applied [2]. The storage and management of tea factory waste as unused waste is subject to legal regulations and is a physically challenging, costly, and labor-intensive process. Some methods applied for waste management (burning, burying, scattering on the ground, dumping into streams and seas) can also cause environmental damage [3,4]. In addition to these applied waste management methods, these products or by-products are utilized in industries such as the production of activated carbon [5], medical products [6,7], caffeine industry [8], ruminant, pig, and poultry feed sector [9,10,11], fish feed industry, and organic fertilizer production [12]. Waste materials that cannot be effectively recycled by factories or utilized as alternatives also have the potential to cause environmental pollution due to their caffeine and phenolic compound content, which can increase soil acidity [3] and alter water quality [13]. In studies [14,15,16] on methane emissions, which cause environmental problems such as global warming, some studies include materials with high levels of tannins and polyphenols, which can be effective on methanogenic archaea, in animal diets. It can be thought that tea factory waste can also be evaluated in this area with its chemical content and properties.

One of the main features that distinguishes tea types from each other is the fermentation process. Among the types of teas, only black teas undergo full fermentation [2]. During black tea production, fermentation and physical processes applied to the plant in factories result in the formation of waste materials in the form of leaf fragments and fibers. These waste materials are known to contain significant amounts of nutrients, including amino acids, non-soluble proteins, sugars, cellulose, polyphenols, flavonols, fiber, tannic acids, zinc, antioxidants, catechins, caffeine, and lignin. Black tea undergoes fermentation with the involvement of enzymes such as polyphenol oxidase, peroxidase, pectinase, alcohol dehydrogenase, transaminase, and peptidase during production [17]. Under the influence of polyphenol oxidase and oxygen, polyphenols, amino acids, carotenoids, and unsaturated fatty acids undergo oxidation [18]. The fermentation process lasts for 3.5 h at a temperature of 26 °C and a relative humidity of 85–95%. After fermentation, the amount of fermented black tea factory waste (FTFW) produced during black tea processing varies based on the production technique [13,19,20].

Studies evaluating the use of tea factory waste in animal nutrition [16,21,22,23,24,25,26,27,28,29] have shown that these wastes can contain varying levels of nutrients, with tannin ranging from 1.8% to 7.89%, dry matter (DM) from 80.88% to 95.0%, crude protein (CP) from 14.0% to 35.0%, crude cellulose (CS) from 14.1% to 18.2%, crude ash (CA) from 3.5% to 7.58%, ether extract (EE) from 1.0% to 7.4%, organic matter (OM) from 88.08% to 94.24%, neutral detergent fiber (NDF) from 38.47% to 47.6%, acid detergent fiber (ADF) from 25.87% to 47.0%, and metabolic energy (ME) values ranging from 7.7 to 19.6 MJ/kg.

The effects of flavonoid derivatives found in the tea plant and tea factory waste, along with their antioxidant, anticarcinogenic, and anti-atherosclerotic properties, have also been referenced in the field of animal nutrition [30]. In this context, data exist on their use in ruminant [31], poultry, pig [32], and fish [33] nutrition, as well as in vitro studies [11,27] and various other applications [34]. These studies indicate that the use of tea factory waste in animal diets may be limited due to factors such as the need to reduce tannin levels for inclusion in small amounts [26] and the potential negative effects on rumen flora [23,31]. Additionally, Konwar et al. [22], Tan [35], and Özyılmaz and Genç [27] have reported that it can be used in limited amounts in different animal diets. However, none of these studies have used rodents. The effects on rodents have mostly been investigated using extracts of different tea types and high doses of pure catechins. Considering the health effects, there is an increasing interest in edible plants containing antioxidants and health-promoting phytochemicals. Tea (*Camellia sinensis*) has antioxidant activity due to its polyphenolic compounds [36,37,38]. Antioxidants neutralize free radicals and reactive molecules that can damage proteins and DNA, and they may prevent the health issues that oxidative stress can cause in the body [39].

This study aims to investigate how different proportions of FTFW in standard rat diets affect overall animal health by analyzing nutritional, histopathological, and biochemical parameters.

## 2. Materials and Methods

### 2.1. Materials

The research material, FTFW, comprises waste generated during the production of tea harvested in May, which is the period exhibiting peak vegetation and nutrient density, from five distinct tea factories located in the Black Sea region. Sampling techniques for feed were applied during the collection of bulk-stored waste [40]. Factory tea wastes were obtained from teas that went through standard processing procedures applied in every tea processing factory in Türkiye [20] and had the same characteristics in terms of cultivation method and period. The study utilized 70-day-old male Wistar albino rats (*n* = 40) weighing 200–250 g as the animal model. After a 1-week acclimatization feeding period, the rats were subjected to feeding for 9 weeks (63 days) at the Ondokuz Mayıs University Experimental Animal Application and Research Center. The total number of rats used in this study was calculated by G*power software (3.1.9.4) based on effect size (0.5), α error probability (0.05), and power (1-β error probability) (0.8) [41]. All animals were housed in plexiglass cages (size 4, LF4 type, 590 × 380 × 200). FTFW was added to standard rat pellet diets at concentrations of 0% (control group), 3%, 5%, and 10% (experimental groups), with each group comprising 10 animals. All diets were isocaloric and isonitrogenous, containing 24% crude protein and 2700 kcal/kg of metabolic energy. These diets were prepared by the ARDEN Research & Experiment Company to meet the standard rat diet contents and to include FTFW. Specific ingredients used in the pellet diet were soybean meal, boncalite, corn, rice bran, vegetable oil, molasses, calcium carbonate, dicalcium phosphate, vitamin/mineral premix, salt, toxin binder, mold inhibitor, and yeast.

### 2.2. Methods

The diets consumed by all groups and FTFW were analyzed for dry matter (DM), crude protein (CP), crude ash (CA), crude cellulose (CS), and nitrogen-free extract (NFE) according to Weende analysis [42]. Ether extract (EE) was quantified using the Soxhlet extraction apparatus based on the method reported by Keskin [43]. The metabolic energy (ME) value was determined according to the formula provided by Yalçın [44]. Total polyphenol analysis was performed using UV spectrophotometry per ISO 14502-1:2005 [45], total catechin content was measured by HPLC according to ISO 14502-2:2005 [46], and caffeine and gallic acid content were measured using UV spectrophotometry in accordance with TS ISO 107207 [47] and TS ISO 10720 [48]. These analyses were conducted at the Rize Tea Research and Application Center (ÇAYMER) Laboratories. Diets and water were provided ad libitum to the animals. During the research period, the animals were housed in Plexiglas cages under conditions of 22 ± 3 °C temperature, 50–60% humidity, and a 12-h light/12-h dark cycle. The body weights of the animals in each group were recorded weekly.

### 2.3. The Preparation of Tissues for Histopathologic Analysis

The animals were euthanized by cardiac exsanguination with a syringe after anesthesia and during surgery. During systematic necropsy, all internal organs were collected, weighed, and subjected to histopathological evaluation. The discussion section focuses on the stomach, kidneys, liver, and intestines, which are the organs most likely to be affected in the digestive system. During necropsy, tissues placed in a 10% buffered formalin solution were fixed for 48 h. To neutralize the effects of formalin, the tissue cassettes were washed under running tap water for 8 h. The cassettes were then processed through a graded series of alcohols (50%, 70%, 80%, 96%, and absolute) and xylene in a tissue processor (Leica^®^, TP1020, Nussloch, Germany). Paraffin blocks were prepared using a tissue embedding unit (Leica^®^, EG1150, Nussloch, Germany). Sections of 5 microns (μm) thickness were cut from the paraffin-embedded tissues using a microtome (Leica^®^, RM2125 RTS, Nussloch, Germany). The tissue sections were stained with hematoxylin and eosin (H&E) [49]. The stained sections were evaluated using a light microscope equipped with a camera (Nikon, Eclipse E600, Tokyo, Japan), and photographs were captured with the Nikon digital-sight imaging system (Nikon DS Camera Head DS-5M, Tokyo, Japan). Histopathological evaluation criteria are shown in Table 1.

### 2.4. Biochemical Analyses

Blood samples were obtained from the hearts of rats after anesthesia and centrifuged at 1550× *g* for 10 min to obtain serum. Glucose, total protein, albumin, triglyceride, total cholesterol, low-density lipoprotein (LDL), high-density lipoprotein (HDL), total bilirubin levels and alanine aminotransferase (ALT), aspartate aminotransferase (AST), alkaline phosphatase (ALP), gamma-glutamyl transferase (GGT) and lactate dehydrogenase (LDH) activities were measured in the serum samples using analyzer kits on an autoanalyzer (A15, Biosystems, Barcelona, Spain). The procedure recommended by the manufacturer was followed to measure biochemical parameters.

### 2.5. Total Antioxidant Status, Total Oxidant Status, and Oxidative Stress Index (OSI) Assays

Total antioxidant status (TAS) [50] and total oxidant status (TOS) [51] were measured using commercially available kits (Rel Assay Diagnostic, Ankara, Türkiye) according to the manufacturer’s instructions. TAS results were expressed as mmol Trolox equivalent/L, and TOS results were expressed in terms of micromolar hydrogen peroxide equivalent per liter (μmol H_2_O_2_ equivalent/L).

OSI was calculated using the following formula:

OSI (arbitrary units) = [(TOS, μmol H_2_O_2_ equivalent/L)/(TAS, μmol Trolox equiva-lent/L) × 100] [52].

### 2.6. Statistical Analysis

The data obtained from the study were analyzed using the SPSS (Statistical Package for Social Sciences, (SPSS Statistics V21.0, IBM Corporation, Armonk, NY, USA) 21.0 statistical analysis program. Skewness and kurtosis values were examined to investigate the conformity of the data to normal distribution. The homogeneity of the data was tested using the Levene Homogeneity test. One-Way Analysis of Variance (One-Way ANOVA) was used to analyze the data that were compatible with normal distribution. Multiple comparisons of the data that were compatible with normal distribution were performed with the Duncan test. *p* < 0.05 was accepted as the significance criterion in all analyses used.

## 3. Results

The study was approved by the Ondokuz Mayis University Animal Experiments Local Ethics Committee under the ethical approval report number 2023-94. Throughout the study, no behavioral changes, differences in feeding and drinking activities, body weight loss, abnormal activities, or mortality were observed in any of the groups. The total polyphenol content of FTFW and rations, total polyphenol in dry matter, gallic acid, caffeine, epicatechin gallate, dry matter, crude protein, ether extract, crude ash, crude cellulose, and metabolic energy values are presented in Table 2.

The amounts of diet (g) consumed by the groups during the research process were recorded as 16,452 g, 16,496 g, 16,488 g, and 16,540 g for the control group (0%) and the diets containing 3%, 5%, and 10% tea factory waste, respectively. The relative weights of internal organs (g/g live weight) and their lengths (cm) are presented in Table 3.

No statistical differences were observed between the groups in terms of relative internal organ weights (*p* > 0.05). The weekly live weight changes of the research groups are presented in Table 4.

### 3.1. Biochemical Findings

Serum glucose, total protein, albumin, triglyceride, total cholesterol, HDL cholesterol, LDL cholesterol, total bilirubin levels, and the activities of AST, ALT, ALP, GGT, and LDH in the groups are presented in Table 5.

No statistical differences were observed between the groups in terms of serum biochemical findings (*p* > 0.05). Although no statistical difference was found, it was observed that glucose levels of group 1 and group 3 decreased and group 2 increased when compared to the control group. Total protein levels tended to decrease in groups 1, 2, and 3 compared to the control group. Albumin levels in the control and group 3 were similar, while those in groups 1 and 2 were slightly lower. Total cholesterol and HDL-cholesterol levels of group 1 tended to decrease slightly compared to the other groups. In group 2, LDL cholesterol levels tended to increase. When compared to the control, it was observed that total bilirubin levels tended to increase in group 2 and decrease in groups 1 and 3. AST activities tended to decrease in groups 1, 2, and 3 compared to healthy controls. ALP activity tended to decrease in group 1 and increase in groups 2 and 3 compared to healthy controls. LDH activity tended to increase in other groups compared to the control group.

### 3.2. Serum TAS, TOS, and OSI Levels

**Figure 1:**
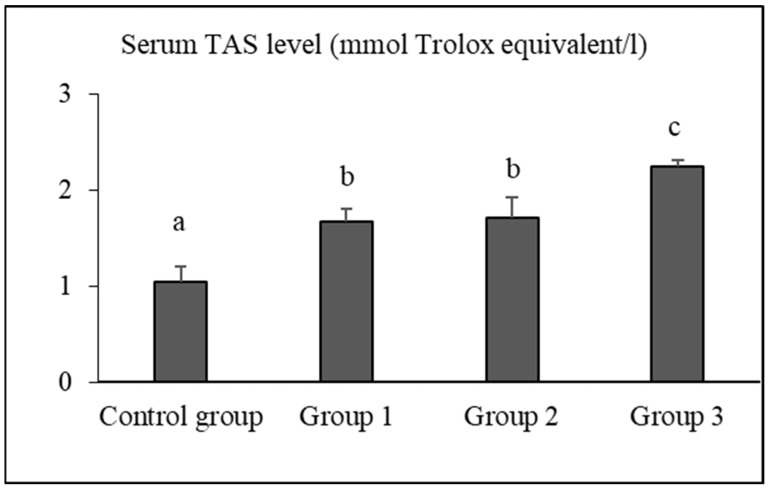
Serum TAS levels of the groups. Different letters (a,b,c) above the bars indicate statistically significant differences. (*p* < 0.05, Tamhane test).

**Figure 2:**
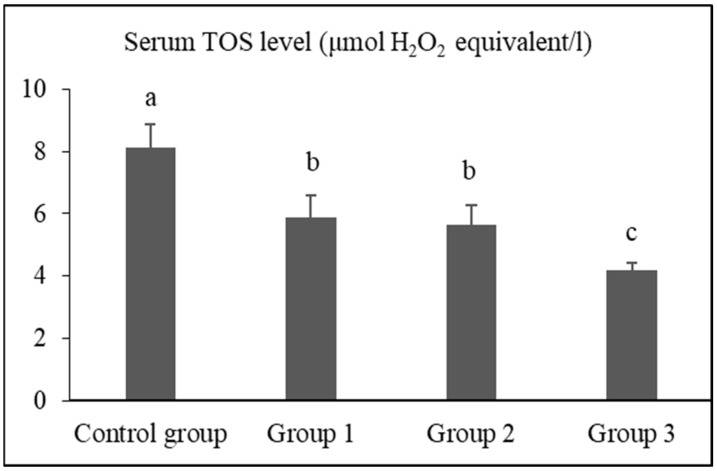
Serum TOS levels of the groups. Different letters (a,b,c) above the bars indicate statistically significant differences (*p* < 0.05, Tamhane test).

**Figure 3:**
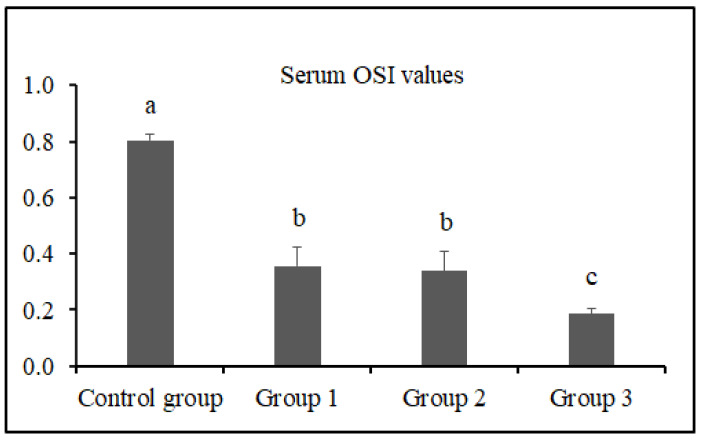
Serum OSI values of the groups. Different letters (a,b,c) above the bars indicate statistically significant differences (*p* < 0.05, Tamhane test).

The serum TAS, TOS, and OSI levels of the groups are presented in Figure 1, Figure 2 and Figure 3, respectively.

The serum TAS levels of rats in the control group, group 1, group 2, and group 3 were determined as 1.04 ± 0.15 mmol Trolox equivalent/L, 1.67 ± 0.12 mmol Trolox equivalent/L, 1.71 ± 0.20 mmol Trolox equivalent/L, and 2.24 ± 0.07 mmol Trolox equivalent/L, respectively. The TAS level of group 3 was significantly higher than that of the control group, group 1, and group 2 (*p* < 0.05).

The serum TOS levels of rats in the control group, group 1, group 2, and group 3 were determined as 8.10 ± 0.76 μmol H_2_O_2_ equivalent/L, 5.86 ± 0.73 μmol H_2_O_2_ equivalent/L, 5.65 ± 0.60 μmol H_2_O_2_ equivalent/L, and 4.17 ± 0.27 μmol H_2_O_2_ equivalent/L, respectively. The TOS level of group 3 was significantly lower than that of the control group, group 1, and group 2 (*p* < 0.05).

The serum OSI values of rats in the control group, group 1, group 2, and group 3 were determined as 0.81 ± 0.02, 0.36 ± 0.07, 0.34 ± 0.07, and 0.19 ± 0.02, respectively. The OSI value of group 3 was significantly lower than that of the control group, group 1, and group 2 (*p* < 0.05).

### 3.3. Pathological Findings

**Figure 4:**
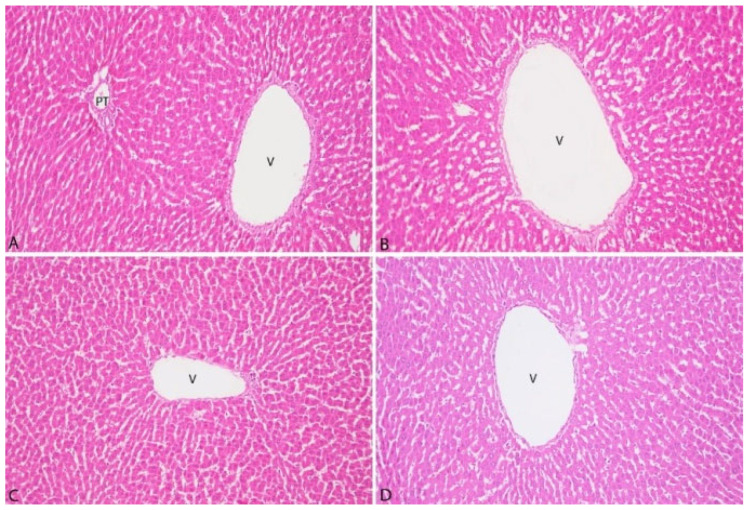
Microphotographs of rat liver tissue in each group (**A**): Control group, (**B**): 3% group, (**C**): 5% group, (**D**): 10% group). V: Vena Centralis, PT: Portal Triad, H&E, original magnification ×200.

The histopathological examination of liver tissues is presented in Figure 4.

The histopathological examination of liver tissues from all groups demonstrated the absence of bile pigments in hepatocytes. Furthermore, no evidence of vacuolar degeneration, microvesicular steatosis, or macrovesicular steatosis was observed. Fibrosis and inflammatory cell infiltration in the portal areas were not detected. Additionally, no signs of congestion were noted in the central veins or sinusoids.

**Figure 5:**
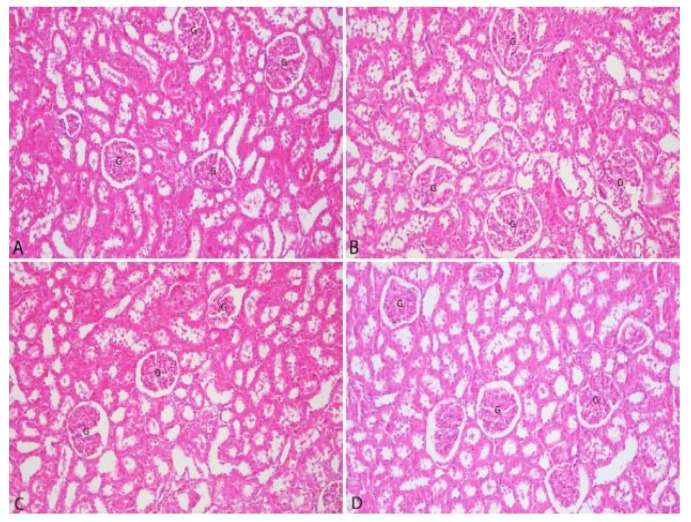
Microphotographs of rat kidney tissue in each group (**A**): Control group, (**B**): 3% group, (**C**): 5% group, (**D**): 10% group. G: Glomerulus, H&E, original magnification ×200.

The histopathological examination of kidney tissues is presented in Figure 5.

The histopathological examination of kidney tissues from all groups revealed no evidence of degeneration in the proximal or distal tubules. Furthermore, hypercellularity and sclerotic changes in the glomeruli were not observed. Additionally, interstitial inflammatory cell infiltrations were not detected in any group.

**Figure 6:**
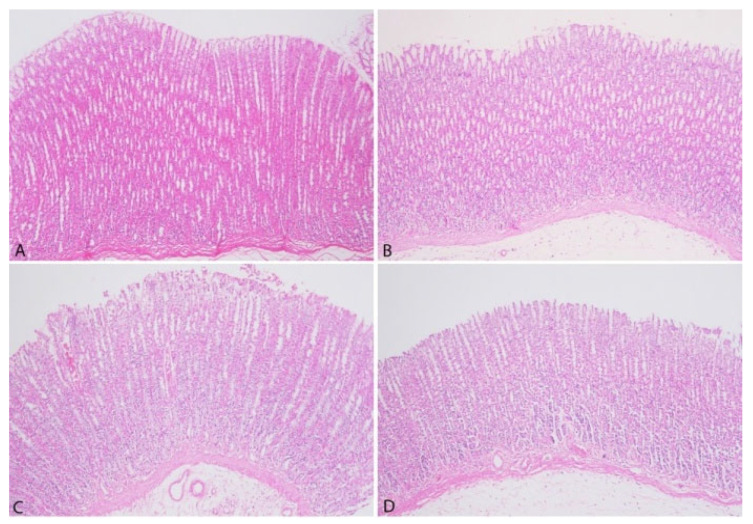
Microphotographs of rat gastric tissue in each group (**A**): Control group, (**B**): 3% group, (**C**): 5% group, (**D**): 10% group. H&E, original magnification ×100.

The histopathological analysis of gastric tissues is presented in Figure 6.

The histopathological analysis of gastric tissues from all groups demonstrated the absence of desquamation in the lamina epithelium. Moreover, no evidence of edema or fibrosis was identified in the submucosa across any group. Lymphatic dilatation within the submucosa was not observed, and no inflammatory cell infiltration was detected in the lamina propria.

**Figure 7:**
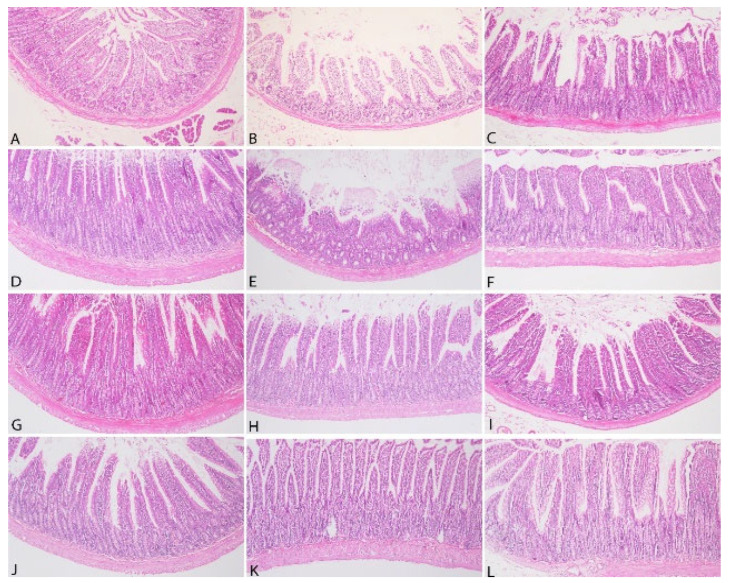
Microphotographs of rat intestinal tissue in each group (**A**): Control group—Duodenum, (**B**): Control group—Jejunum, (**C**): Control group—Ileum, (**D**): 3% group—Duodenum, (**E**): 3% group—Jejunum, (**F**): 3% group—Ileum, (**G**): 5% group—Duodenum, (**H**): 5% group—Jejunum, (**I**): 5% group—Ileum, (**J**): 10% group—Duodenum, (**K**): 10% group—Jejunum, (**L**): 10% group—Ileum. H&E, original magnification ×100.

The histopathological analysis of intestinal tissues is presented in Figure 7.

The histopathological analysis of intestinal tissues from all groups demonstrated the absence of desquamation in the lamina epithelium. No lymphatic dilatation was observed in the submucosa, and hyperplasia of the Lieberkühn glands was not detected. However, mononuclear inflammatory cell infiltrations, primarily composed of lymphocytes, were identified in the lamina propria in all groups.

## 4. Discussion

Contrary to expectations that tea factory waste would be unpalatable due to its caffeine and tannin content, potentially causing constipation and reducing feed intake, our study found no evidence to support this hypothesis. Such assumptions and associated effects are more commonly observed in ruminant nutrition research [23,29,31]. In our study, the caffeine content naturally present in FTFW was significantly reduced during the pelleting process. Additionally, theanine constitutes 50% of the amino acid composition of fermented tea plants. This amino acid has an aromatic effect and is also found in tea factory waste [53], suggesting that it may contribute to enhancing palatability. The formation of aromatic compounds varies not only with the processing procedures of tea but also with the harvest time in nature [54]. The FTFW used in this study was sourced from tea plants harvested in May, an optimal period in terms of nutrient content and aroma. Consistent feed intake across all groups and the stable live weight gain throughout the study period (*p* > 0.05) corroborate this finding. Furthermore, no defecation issues related to constipation were observed in any group, and necropsy findings revealed no signs of constipation-related abnormalities in the intestines. Wu et al. [55] demonstrated that fermented black tea extract alleviated induced constipation symptoms in mice and was associated with a reduction in colonic AQP3 and AQP9 protein expression. The constipation-preventing effect and the acceleration of gastric and intestinal content transit in mice have been reported to be attributed to active compounds found in fermented tea plants, including saponins, thearubigins (TRs), soluble sugars, tea polysaccharides (TPSs), gallic acid (GA), and catechin gallate (CG) [55,56]. Although there are a limited number of studies investigating the toxicity of black [57], green [58], and Pu-erh [59] tea extracts in rodents, no research has been found demonstrating the effects of FTFW.

Sur et al. [57] reported that oral administration of black tea infusion extract for 90 days did not cause any toxicity in Swiss albino mice at doses up to 2 g/kg and in Wistar albino rats at doses up to 250 mg/kg/day. They observed that body weight, food intake, hematological parameters, serum biochemistry, and organ weights were not adversely affected. Additionally, no pathological or abnormal findings were detected in the organs, skin, or fur, and no behavioral changes were observed. These findings, which used black tea extract, the material closest to the FTFW used in our research, align closely with our study results.

Al-Attar and Zari [60] reported that crude tea leaf extract reduced body weight loss in diabetic mice and positively influenced body weight gain in healthy animals after the 30th day. In the same study, it was observed that glucose, triglyceride, cholesterol, total protein, GPT, and GOT levels showed a lower increase in diabetic animals, while in non-diabetic animals, the values were similar to those of the control group, which is consistent with the findings of our study. Salem et al. [61], in agreement with our study, reported that green tea extract did not affect AST, ALT, TSB, and albumin levels in rats. Additionally, in antioxidant capacity tests, they found that superoxide dismutase (SOD), catalase (CAT), malondialdehyde (MDA), and glutathione (GSH) levels were similar to those in the control group.

In a study on the effect of green tea on oxidative stress, Azeez et al. [62] evaluated the protective effect of green tea (GT) against hydrogen peroxide-induced oxidative stress in rats. It was reported that GT reduced cholesterol, triglyceride, LDL, and MDA levels, increased HDL levels, and protected against lipid peroxidation. Studies have indicated that caffeine in tea extracts may induce CYP1A2, a CYP450 derivative, which is associated with tissue toxicity [63,64,65]. In our study, after FTFW was incorporated into the pelleted diet, caffeine levels remained at only a very low concentration (0.09 ppm) in the 10% group and were completely eliminated in the other groups. Consequently, even at a 10% inclusion level, there was no risk of caffeine-induced toxicity or behavioral changes. The reduction in caffeine levels suggests the possibility of denaturation due to heat (80–85 °C), moisture (10–15%), and pressing during the pellet production process.

Chen et al. [66] and Li et al. [67] reported that the inclusion of tea flowers, which have a chemical composition similar to FTFW and are also considered industrial waste, in a standard diet did not lead to any mutagenicity, acute or subchronic toxicity, hematological abnormalities, body weight alterations, or organ pathologies in rats.

Takami et al. [68] administered green tea catechins (GTC) at levels of 0.3%, 1.25%, and 5.0% orally to F344 rats for 90 days. In the high catechin group, body weight loss and an increase in albumin/globulin ratio (A/G), alanine transaminase (ALT), and aspartate transaminase (AST) were observed. The changes in A/G, ALT, and ALP levels were suggested to be related to the mild hepatotoxic effects of GTC. Additionally, total cholesterol and triglyceride levels were found to decrease in the experimental groups. In the high-dose GTC group (0.5%), the weights of the brain, thymus, lungs, heart, spleen, liver, pituitary gland, adrenal glands, and thyroid were significantly lower (*p* < 0.05). However, these effects, observed with the administration of pure GTC, were not observed in our study with FTFW-supplemented pelleted diets.

Gür et al. [69] found that long-term use of black tea extract protected plasma proteins and lipids as well as liver and kidney tissues against oxidative damage. It is well known that these protective effects are primarily attributed to flavonoids, which belong to the polyphenol group [70]. Unlike previous studies reporting the antioxidant effects of black tea extract in plasma [69,70,71], our study demonstrated that the TAS values were highest (*p* < 0.05), and TOS and OSI values were lowest (*p* < 0.05) in the 10% FTFW group. These findings indicate that similar antioxidant effects can be achieved by using FTFW instead of tea extract, and this effect is thought to be attributed to the polyphenols present in FTFW. The mechanisms behind the antioxidant effects of polyphenols in tea are known to be neutralizing radicals, chelating metals, inhibiting prooxidative enzymes, and suppressing the consumption of intracellular antioxidant enzymes [72,73]. Black tea polyphenols have been observed to upregulate antioxidant enzymes, including quinone oxidoreductase 1 and glutathione S-transferase, in mouse liver and lung via the Nrf2-ARE pathway [74]. In this study, tea factory waste added to rat feed suppressed oxidative stress and OSI, depending on the dose, with the strongest effect observed in the group given 10%. The observation that serum TOS level decreased 1.38-fold in the 3% FTFW group, 1.43-fold in the 5% FTFW group, and 1.94-fold in the 10% FTFW group compared to the control group indicates that systemic oxidative stress was suppressed with the increase in the amount of factory tea waste added to the rat diet.

The fact that serum TAS levels in rats fed FTFW increased in parallel with the increase in the ratio in the diet and that the highest fold increase (2.15) was found in rats fed 10% FTFW suggests that higher levels of polyphenols are responsible for this effect. Tea polyphenols have been reported to ameliorate oxidative stress induced by hydrogen peroxide and constant darkness by regulating the Keap1/Nrf2 transcriptional signaling pathway in HepG2 cells and mouse liver [75]. Considering that a diet rich in antioxidants reduces the risk of many diseases, delays aging, and improves quality of life, it is obvious that the addition of FTFW, which has proven antioxidant effects, to the rat diet will provide significant health benefits. Adding FTFW to the diet of mice is likely to have the same beneficial effects as in rats, but this needs to be investigated.

Although catechins found in the tea plant are generally considered non-toxic in mice and rats, studies have reported cases of erythema and papular lesions on the skin, conjunctivitis, scleritis, chemosis, discharge, enlarged colon, hemorrhagic fluid in the small intestine, toxicity, hepatic necrosis, skin tumors, and glutathione S-transferase placental form carcinogenesis in the liver [58,76,77,78,79,80]. Additionally, convulsive effects have also been reported by Gomes et al. [81]. In our study, none of these symptoms were observed in any of the groups fed with FTFW. As emphasized in the studies by Hirose et al. [77] and Yamane et al. [82], differences in research outcomes may arise due to the non-standardized catechin derivatives in tea plants. It can be concluded that the effects observed with pure and highly concentrated catechins in extract form are unlikely to occur when FTFW is incorporated into pelleted diets at levels up to 10%.

Although green tea has been reported to be safely used in rat diets at doses of 764 mg/kg BW/day for males and 820 mg/kg BW/day for females, Takami et al. [68] and Chan et al. [65] observed that at doses ≥ 250 mg/kg, there was a decrease in total protein and albumin levels, along with ruffled fur, necrotic hepatocytes, hemorrhage, and moderate centrilobular necrosis characterized by mononuclear infiltration in the centrilobular regions. In mice, high-dose consumption was associated with increased activity, hepatic necrosis-induced steatosis, glycogen depletion, pigment accumulation, increased mitosis, karyomegaly, chronic inflammation, and early mortality. Additionally, lethargy, abnormal breathing, ataxia, respiratory epithelial atrophy, splenic and mesenteric lymphoid atrophy, and reduced body weight were observed. However, hematological parameters remained unaffected by the treatment. Although the exact cause of body weight reduction and changes in feeding behavior is not fully understood, these effects have been associated with inhibition of lipid absorption and stimulation of lipid oxidation. In our study, none of the pathological findings induced by green, black, and oolong tea extracts or pure catechin derivatives were observed in any group. In studies where pathological effects were detected, it was observed that the chemicals contained in the tea plant were applied in purified extract forms and at high (potentially toxic) doses. The fact that no pathological effects were encountered in our study can be attributed to the use of natural and herbal waste, the enzymatic reactions that occur during tea production, the preservative effects that occur with fermentation, and the caffeine denaturation that may occur during the pelleting process.

The strength of the study is that it shows that FTFWs can be safely included in rat diets without causing any pathological findings or health and developmental problems, not only with animal feeding parameters but also with the use of both biochemical and histopathological analyses. In addition, it should not be forgotten that the time-dependent conduct of in vivo studies is a limiting factor in making an assessment from a broader perspective and determining the physiological and metabolic effects that will occur in the long term. Therefore, new studies may be required, including in vitro tests, physical property tests of pellets, and the use of animals from different strains, which also involve evaluations in terms of efficacy and economics.

## 5. Conclusions

While the literature predominantly reports numerous cases of acute and chronic pathological conditions, as well as abnormal biochemical parameters, associated with the use of green and black tea extracts in various animal species, these effects do not appear to extend to fermented black tea factory waste (FTFW) used in rat diets. Its antioxidant properties and protective effects may offer a potential advantage in conventional laboratory animal husbandry. The findings suggest that fermented tea factory waste can be incorporated into rat diets without causing changes in body weight gain, feed intake, or overall health status. Furthermore, this study highlights the potential to convert a material that would otherwise contribute to environmental pollution into a beneficial, low-cost, and antioxidant-rich product, thereby supporting economic sustainability. It appears worthy of investigation to assess the feasibility and economic viability of using FTFW on a larger scale. It is recommended that similar studies be conducted on other monogastric laboratory animal species and diets to further explore the applicability of these findings.

## Figures and Tables

**Table 1:** Histopathological Evaluation Criteria.

Liver	Kidney
Bile Pigment in Hepatocytes	Proximal Tubular Epithelium Degeneration
Vacuolar Degeneration	Distal Tubular Epithelium Degeneration
Macrovesicular Steatosis	Crystalluria
Microvesicular Steatosis	Hypercellularity in Glomeruli
Inflammatory Cell Infiltration in the Portal Area	Glomerulosclerosis
Portal Area Fibrosis	Expansion of Bowman’s Space
Central Vein Congestion	Inflammatory Cell Infiltration in Interstitial Tissue
Congestion in the Sinusoids	
**Stomach**	**Intestine**
Lamina Epithelium Desquamation	Lamina Epithelium Desquamation
Inflammatory Cell Infiltration in Lamina Propria	Inflammatory Cells in Lamina Propria
Submucosal Edema	Lieberkühn Glands Hyperplasia
Submucosal Fibrosis	Lymphatic Dilatation

**Table 2:** The values of total polyphenol (TP, ppm), total polyphenol in dry matter (TPDM, ppm), gallic acid (GA, ppm), caffeine (ppm), epicatechin gallate (ECG, ppm), dry matter (DM, %), crude protein (CP, %), ether extract (EE, %), crude ash (CA, %), crude cellulose (CC, %), and metabolic energy (ME, kcal/kg) in FTFW and rations (0%, 3%, 5%, 10%).

Parameters	Groups				
	FTFW	C 0%	1 (3%)	2 (5%)	3 (10%)
TP (ppm)	5.35	1.19	1.20	1.38	1.42
TPDM (ppm)	1.72	n	n	n	0.10
GA (ppm)	0.10	n	n	n	n
Caffeine (ppm)	1.17	n	n	n	0.09
ECG (PPM)	0.28	n	n	n	n
DM (%)	92.00	93.00	93.20	92.90	93.00
CP (%)	14.00	24.00	24.00	24.00	24.00
EE (%)	1.20	4.00	4.00	4.00	4.00
CA	3.50	8.40	8.38	8.41	8.40
CC	18.00	6.00	6.02	6.03	6.05
ME (kcal/kg)	2400	2700	2700	2700	2700

C: Control group, TP: Total polyphenol, TPDM: Total polyphenol in dry matter, GA: Gallic acid, ECG: Epicatechin gallate, DM: Dry matter (%), CP: Crude protein, EE: Ether extract, CA: Crude ash, CC: Crude cellulose, ME: Metabolic energy, n: Not detected.

**Table 3:** Relative weights of internal organs (g/g live weight) and their lengths (cm) (Mean ± SE) (n = 10).

Groups	Internal Organs										
	Heart (g)	Liver (g)	Lung (g)	Stomach (g)	Spleen (g)	Kidney (g)	Brain (g)	Small Intestine (g)	Large Intestine (g)	Small Intestine Length (cm)	Large Intestine Length (cm)
C (0%)	1.22 ± 0.05	10.22 ± 0.38	2.02 ± 0.13	1.78 ± 0.09	0.66 ± 0.03	2.97 ± 0.09	1.49 ± 0.11	9.52 ± 0.48	4.17 ± 0.36	120.20 ± 3.06	21.40 ± 1.39
1 (3%)	1.30 ± 0.03	11.24 ± 0.37	2.24 ± 0.12	1.83 ± 0.05	0.64 ± 0.02	3.05 ± 0.12	1.60 ± 0.10	9.79 ± 0.41	4.45 ± 0.30	128.60 ± 2.80	19.30 ± 1.00
2 (5%)	1.23 ± 0.03	10.91 ± 0.46	2.18 ± 0.08	1.86 ± 0.08	0.70 ± 0.02	3.08 ± 0.18	1.45 ± 0.11	9.67 ± 0.36	4.48 ± 0.32	122.80 ± 2.86	19.70 ± 0.59
3 (10%)	1.27 ± 0.04	11.23 ± 0.38	2.10 ± 0.10	1.96 ± 0.07	0.63 ± 0.02	2.98 ± 0.09	1.41 ± 0.10	9.85 ± 0.36	4.85 ± 0.22	121.40 ± 3.67	21.50 ± 1.20
*p*-value	0.505	0.257	0.537	0.384	0.345	0.918	0.652	0.942	0.500	0.252	0.365

C: Control group.

**Table 4:** Weekly live body weight (g) (mean ± SE) changes of the research groups (n = 10).

Groups	Week									
	First Day	1.	2.	3.	4.	5.	6.	7.	8.	9.
C (0%)	218.70 ± 7.03	261.10 ± 10.00	298.10 ± 12.26	324.60 ± 13.55	345.10 ± 13.37	367.20 ± 13.89	384.80 ± 14.46	396.70 ± 14.45	415.60 ± 15.72	422.60 ± 15.83
1 (3%)	219.10 ± 7.18	270.10 ± 8.28	309.70 ± 10.12	338.50 ± 11.08	365.30 ± 11.45	391.30 ± 12.28	409.70 ± 13.34	424.80 ± 12.88	442.30 ± 14.00	449.60 ± 14.07
2 (5%)	217.70 ± 5.52	261.50 ± 9.35	300.00 ± 9.81	328.70 ± 10.60	354.60 ± 10.58	377.40 ± 11.15	394.60 ± 11.95	407.40 ± 12.99	422.50 ± 13.65	432.40 ± 14.21
3 (10%)	218.80 ± 8.66	267.60 ± 9.95	299.80 ± 10.66	335.30 ± 11.46	358.80 ± 10.12	381.80 ± 9.94	400.90 ± 10.87	413.00 ± 10.34	430.10 ± 9.78	439.20 ± 10.67
*p*-value	0.999	0.878	0.868	0.834	0.654	0.555	0.571	0.480	0.546	0.543

C: Control group. No statistical differences were observed between the groups in terms of weekly live body weight findings (*p* > 0.05).

**Table 5:** Serum biochemical findings of the groups. (n = 10).

Parameter	Groups			
	C (0%)	1 (3%)	2 (5%)	3 (10%)
Glucose (mg/dL)	129.4 ± 25.0	128.8 ± 20.1	130.1 ± 24.4	126.7 ± 20.2
Total protein (g/L)	64.2 ± 3.1	62.1 ± 4.1	61.5 ± 3.4	61.4 ± 2.9
Albumin (g/L)	30.5 ± 1.5	29.4 ± 2.3	29.8 ± 1.6	30.6 ± 1.4
Triglyceride (mg/dL)	43.8 ± 12.9	34.4 ± 11.5	40.1 ± 12.8	38.1 ± 11.4
Total cholesterol (mg/dL)	50.8 ± 8.4	49.9 ± 8.6	51.1 ± 8.2	52.0 ± 11.0
HDL cholesterol (mg/dL)	26.8 ± 4.5	25.2 ± 3.3	27.7 ± 2.7	29.9 ± 3.8
LDL cholesterol (mg/dL)	13.3 ± 1.2	13.2 ± 1.0	14.1 ± 0.9	13.4 ± 1.3
Total bilirubin (mg/dL)	0.54 ± 0.35	0.43 ± 0.24	0.57 ± 0.21	0.44 ± 0.31
AST (U/L)	65.8 ± 10.6	57.3 ± 7.4	59.6 ± 13.3	59.8 ± 9.8
ALT (U/L)	28.9 ± 5.8	27.1 ± 4.6	29.3 ± 5.2	28.2 ± 2.1
ALP (U/L)	127.9 ± 32.9	120.2 ± 23.0	133.1 ± 32.4	135.8 ± 29.5
GGT (U/L)	3.5 ± 0.5	3.7 ± 0.7	3.3 ± 0.7	3.9 ± 0.7
LDH (U/L)	241.1 ± 20.2	253.0 ± 23.9	261.6 ± 27.7	254.3 ± 32.6

C: Control group. HDL: High-Density Lipoprotein, LDL: Low-Density Lipoprotein, AST: Aspartate Aminotransferase, ALT: Alanine Aminotransferase, ALP: Alkaline Phosphatase, GGT: Gamma-Glutamyl Transferase, LDH: Lactate Dehydrogenase.

## Data Availability

The datasets produced and examined during this study can be accessed from the corresponding author upon reasonable request.
